# Harvesting Energy from Bridge Vibration by Piezoelectric Structure with Magnets Tailoring Potential Energy

**DOI:** 10.3390/ma15010033

**Published:** 2021-12-21

**Authors:** Zhiyong Zhou, Haiwei Zhang, Weiyang Qin, Pei Zhu, Ping Wang, Wenfeng Du

**Affiliations:** 1 School of Civil Engineering and Architecture, Henan University, Kaifeng 475004, China; 10160091@vip.henu.edu.cn (Z.Z.); hwzhang@henu.edu.cn (H.Z.); 10160016@vip.henu.edu.cn (W.D.); 2 School of Power and Energy, Northwestern Polytechnical University, Xi’an 710072, China; 3 Department of Engineering Mechanics, Northwestern Polytechnical University, Xi’an 710072, China; qinweiyang@nwpu.edu.cn; 4 AECC Hunan Aviation Powerplant Research Institute, Zhuzhou 412002, China

**Keywords:** energy harvesting, bridge vibration, vehicle, mono-stable energy harvester, linear energy harvester, moving speed

## Abstract

Bridges play an increasingly more important role in modern transportation, which is why many sensors are mounted on it in order to provide safety. However, supplying reliable power to these sensors has always been a great challenge. Scavenging energy from bridge vibration to power the wireless sensors has attracted more attention in recent years. Moreover, it has been proved that the linear energy harvester cannot always work efficiently since the vibration energy of the bridge distributes over a broad frequency band. In this paper, a nonlinear energy harvester is proposed to enhance the performance of harvesting bridge vibration energy. Analyses on potential energy, restoring force, and stiffness were carried out. By adjusting the separation distance between magnets, the harvester could own a low and flat potential energy, which could help the harvester oscillate on a high-energy orbit and generate high output. For validation, corresponding experiments were carried out. The results show that the output of the optimal configuration outperforms that of the linear one. Moreover, with the increase in vehicle speed, a component of extremely low frequency is gradually enhanced, which corresponds to the motion on the high-energy orbit. This study may give an effective method of harvesting energy from bridge vibration excited by moving vehicles with different moving speeds.

## 1. Introduction

Bridge safety has drawn great concern in recent decades owing to the developments in the cross-sea bridge, cable-stayed bridge, and suspension bridge [1]. To ensure the safety of the bridge, a sensor network should be incorporated into it so that many node sensors are distributed to monitor the state of the bridge to prevent hazards caused by degradation [2]. For the node sensors, the replacement of the battery is a difficult challenge [3]. Frequent battery replacement is excessively expensive and nearly impossible in some dangerous and special hard-to-reach areas [4]. To tackle the difficulty, harvesting energy from bridge vibration to power sensors is emerging as a promising solution. In principle, the vibration energy of structure can be converted to electric energy through three methods, i.e., the triboelectric, electro-magnetic, and piezoelectric mechanisms [5].

The piezoelectric scenario generally scavenges energy from bridge vibration excited by travelling vehicles. Developing energy harvesting devices coupled with the bridge structure has been drawing more and more attention from scholars [6]. Assadi et al. [7] attached a piezoelectric patch to a simply supported beam with a mass moving on it. When the moving mass travelled on the beam, the piezoelectric patch could produce voltage due to deflection. The analytical and experimental results indicated that the speed of the moving mass had a significant influence on voltage output. Ali et al. [8] used a linear single-degree-of-freedom model as an energy harvester to harvest the vibration energy of a simply supported beam excited by the motion of a point load. Galchev et al. [9] designed an inertial energy harvester to convert traffic-induced bridge vibrations to electric energy. The average power of this fabricated device was measured at different positions of a suspension bridge. Peigney and Siegert [10] designed and tested a cantilever piezoelectric harvester to harvest vibration energy from traffic-induced vibrations in bridges. Erturk [11] derived analytical expressions of transient response in the time domain of piezoelectric output power. Zhang et al. [12] conducted a numerical parametric study and derived the exact analytical solution of a piezoelectric energy harvester excited by beam vibration under moving harmonic loads. Amini [13] numerically investigated the harvested power from the vibrations of a beam excited by multi-moving loads. The results showed that the efficiency of the harvester increased with an increase in moving velocity. Xiang et al. [14] investigated a pavement system under moving vehicles to scavenge traffic-induced energy using piezoelectric transducers. When the velocity of a moving vehicle was close to a critical value, the power output of the piezoelectric harvester was optimal. Romero et al. [15] analyzed the energy harvesting performance of railway bridges in different operational conditions. The results showed that the passage of trains had a major effect on energy harvesting performance. Therefore, the typical cantilevered (linear) harvester always had an enormous challenge in scavenging energy from bridge vibration since the abundant vibration energy of a bridge excited by different vehicles exists within a broad frequency band.

At present, harvesting energy from base vibrations is being widely investigated, in which the magnetic or axial loading forces are often used to enhance harvesting performance. Accordingly, the nonlinearity caused by the introduced magnetic forces was studied in depth so as to improve the enhancement in harvesting ability [16]. With the development of the study, the configurations with different kinds of stability, e.g., mono-stability [17], bi-stability, tri-stability [18], quad-stability [19], and even penta-stability, were proposed and proved to be able to broaden the operating bandwidth effectively. For example, Fan et al. [20] proposed a mono-stable piezoelectric energy harvester to improve the efficiency of energy extraction from low-level excitations by two stoppers and four magnets. Naseer et al. [21] theoretically investigated harvesting energy from vortex-induced vibrations by using nonlinear attractive magnetic forces. Jang and Chen [22] derived the Fokker–Plank–Kolmogorov equation of a mono-stable Duffing oscillator with piezoelectric coupling. The effects of the bandwidth, initial conditions, and cubic nonlinearity on voltage were numerically studied. Erturk and Inman [23] conducted the theoretical and experimental studies on the high-energy orbits of a bi-stable energy harvester. Moreover, the relative advantages and trade-offs of bi-stable and mono-stable harvesters were presented by Zhao and Erturk [24]. Although the mono-stable energy harvester is more robust and reliable than the bi-stable one under stochastic excitations, the bi-stable energy harvester should be designed deliberately to work more efficiently for a given excitation. Kim and Seok [25] developed the mathematical model of an energy harvester with multi-stable (mono-, bi-, and tri-stable) characteristics. The results of the simulation showed that the tri-stable energy harvester had a wider bandwidth than both the bi-stable and linear ones. Wang et al. [26] utilized the tri-stable characteristics to enhance the energy harvesting performance of a galloping piezoelectric energy harvester.

In previous studies, the linear vibration energy harvesters had been adopted to scavenge energy from bridge vibration, but it was found that they could not work efficiently under broadband excitations. Hence, nonlinearity was introduced into the design of the energy harvester under different excitation types, e.g., the harmonic, stochastic, or flow-induced vibrations. The multi-stability created by magnetic interaction has been proved to be particularly effective. Striking improvement in energy harvesting performance has been demonstrated. However, in the multi-stable structures, the energy required to cross the potential barrier and produce a big deflection is fairly large. In this study, we tried using nonlinear magnetic forces to obtain an optimal shape for potential energy. To the best of the authors’ knowledge, using magnetic nonlinearity to tailor potential energy has not been studied in the harvesting of bridge vibration energy. These techniques can produce large amplitudes by creating a desired potential energy shape rather than a multi-stable shape. In this paper, a scenario of harvesting the energy of a bridge by mono-stability is proposed, in which the magnetic forces are introduced to form a desirable potential energy and a high-energy orbit. The mono-stable piezoelectric energy harvester (MPEH) can produce a large output if it oscillates in the high-energy orbit. It was proved that the MPEH could reach a high efficiency in harvesting the vibration energy of a bridge. Firstly, for the particular mono-stable characteristic, corresponding analyses on potential energy, restoring force, and stiffness were carried out. The results could help determine the optimal shape for potential energy. Then, experimental studies were conducted, and the dynamic responses and electrical outputs were investigated under different conditions. Finally, a summary is presented and some conclusions are drawn at the end of this paper.

## 2. Energy Harvester for Bridge Vibration Excited by Moving Vehicles

**Figure 1:**
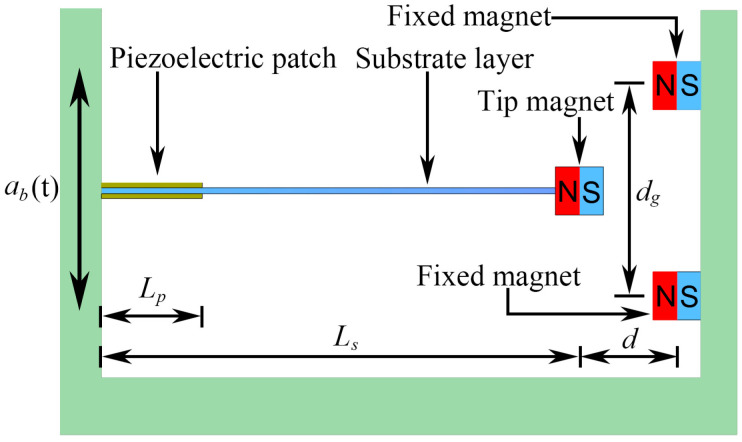
Schematic diagram of MPEH.

The schematic of MPEH is shown in Figure 1, which is composed of a cantilever beam with two piezoelectric patches attached to the root, a tip magnet fixed at the beam’s free end, and two fixed magnets. The three magnets are placed such that the tip and fixed magnets are magnetically attractive. By adjusting the separation distance (*d*) and gap distance (*d_g_)*, the potential energy can be tailored to own a flat-shaped bottom, which is beneficial for the MPEH to execute a large-amplitude vibration. In particular, the directions of the magnetic and elastic forces are opposite to each other, which could move the operating frequency band toward the low frequency, thus reducing the energy required to produce a large deflection of the harvester and improving the voltage and power outputs. The classical linear piezoelectric energy harvester (LPEH) often shows inefficiency since the abundant vibration energy excited by moving vehicles at different speeds exist in the form of a broad frequency band with small magnitude. In order to collect more electric energy, a large LPEH (especially a very long substrate layer with a length $L_{s}$) is required; however, it is difficult to meet the energy supply requirements of the densely distributed sensors. Therefore, scavenging energy from bridge vibration by linear structures has proved to be an enormous challenge. Compared with LPEH, the MPEH can attain the high-energy orbit under the excitation of passing vehicles and generate a high output.

**Figure 2:**
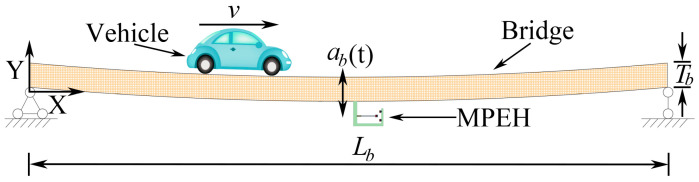
Schematic diagram of energy harvesting from bridge vibration excited by a moving vehicle.

As Figure 2 shows, the MPEH is fixed to a bridge with the length of $L_{b}$ and the thickness of $T_{b}$. Then, as a moving vehicle travels on the bridge at speed v, its load excites the elastic bridge to oscillate with the acceleration $a_{b}\left(t\right)$. Thus, the MPEH oscillates and generates electric power through piezoelectric materials. According to previous related studies, the bridge can be modeled as an Euler–Bernoulli beam, which is a simply supported elastic beam with infinite degrees of freedom. The moving vehicle travelling on this beam with hinged-hinged boundary conditions can be simplified as a moving mass.

## 3. Analysis of Potential Energy, Restoring Force, and Stiffness

The total potential energy of MPEH consists of two parts: the elastic potential energy of the substrate and the magnetic potential energy that originated from the magnets.

According to the Euler–Bernoulli beam theory, the strain of the piezoelectric cantilever is proportional to the second spatial derivative of deflection. Hence, the potential energy calculation in this paper has been based on the following two assumptions: (a) the flat section perpendicular to the center line of the beam before deformation still stays flat after deformation; (b) the plane of the deformed cross section is perpendicular to the deformed axis. Then, the elastic potential energy of the piezoelectric cantilever beam can be given by the following equation [27]:

(1) $$U_{e}=\frac{1}{2}E_{s}I_{s}\int_{0}^{L_{s}}{\left(\frac{\partial^{2}w\left(x,t\right)}{\partial x^{2}}\right)}^{2}dx+E_{p}I_{p}\int_{0}^{L_{p}}{\left(\frac{\partial^{2}w\left(x,t\right)}{\partial x^{2}}\right)}^{2}dx$$

where $E_{s}$ is the Young modulus of the substrate; $I_{s}$ is the inertia moment of the substrate given by $I_{s}=\frac{W_{s}T_{s}^{3}}{12}$ ($W_{s}$ and $T_{s}$ are the width and thickness of the substrate, respectively); $L_{s}$ is the length of the substrate; $E_{p}$ is the bending stiffness of the piezoelectric patch; $I_{p}$ is the total inertia moment of two piezoelectric patches on both sides of the substrate, which can be given by $I_{p}=\frac{W_{p}T_{p}\left(4T_{p}^{2}+6T_{p}T_{s}+3T_{s}^{2}\right)}{6}$ ($W_{p}$ and $T_{p}$ are the width and thickness of the piezoelectric patch, respectively); $L_{p}$ is the length of the piezoelectric patch; w(x,t) is the displacement of the cantilever beam.

The magnetic potential energy can be obtained based on the assumption of a magnetic dipole. Thus, the magnetic potential energy generated by two fixed magnets upon the tip of the magnet can be expressed as follows [28]:

(2) $$U_{m}=-\frac{\mu_{0}a_{1}a_{2}}{2\pi }{\left\{{\left(w\left(L_{s},t\right)-\frac{d_{g}}{2}\right)}^{2}+d^{2}\right\}}^{-\frac{3}{2}}-\frac{\mu_{0}a_{1}a_{3}}{2\pi }{\left\{{\left(w\left(L_{s},t\right)+\frac{d_{g}}{2}\right)}^{2}+d^{2}\right\}}^{-\frac{3}{2}}$$

where $\mu_{0}$ is the permeability constant; $a_{1}$ and $a_{2}$ ($a_{3}$) are the effective magnetic moment of the tip magnet and the fixed magnets, respectively; d is the horizontal distance from the center of the tip magnet to the center of the fixed magnets; $d_{g}$ is the gap distance between the two fixed magnets.

**Figure 3:**
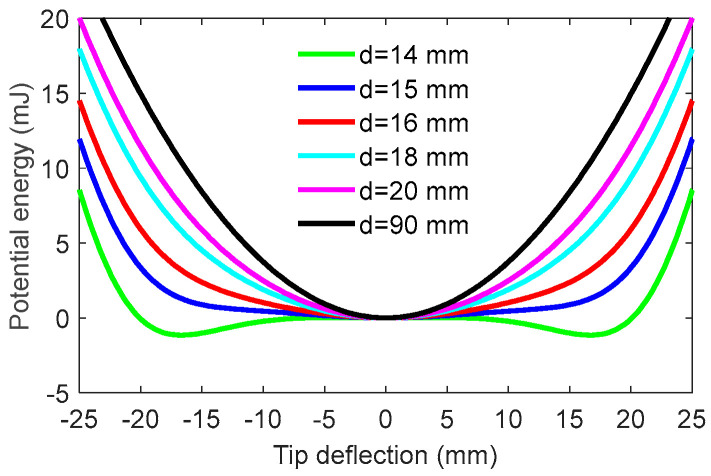
Potential energy for different values of separation distance.

Since the potential energy is determined by the separation distance *d*, the total potential energy is computed with the distance *d* varying so as to illustrate the influence of *d*. The results are illustrated in Figure 3. The system parameters are given in Table 1. It can be seen from Figure 3 that as *d* decreases from 90 mm to 14 mm, the potential energy experiences the linear stable state, the mono-stable state, and then the bi-stable state. Specifically, when *d* is quite large, the system acts nearly as a linear one resulting from a very weak magnetic force. With a decrease in the separate distance *d*, the magnetic force will become large. The potential energy could own a steep bottom, implying that the MPEH may have a relatively large amplitude of vibration under external excitations. The ideal mono-stable configuration is expected to make a strong magnetic coupling between the magnetic force and the elastic force. This desirable case can provide a large beam deflection and then a high-voltage output under low-level bridge excitations. Especially if *d* is smaller than a critical value, i.e., 14 mm, the magnetic force will be larger than the elastic restoring force. With two stable equilibrium positions emerging, the MPEH becomes a bi-stable system. However, in the bi-stable state, two potential wells and a potential barrier will appear, which would need more excitation energy. Thus, for weak excitations, the bi-stable MPEH may not be the best option. In contrast, the mono-stable state near the bi-stable one is the most appropriate choice.

**Figure 4:**
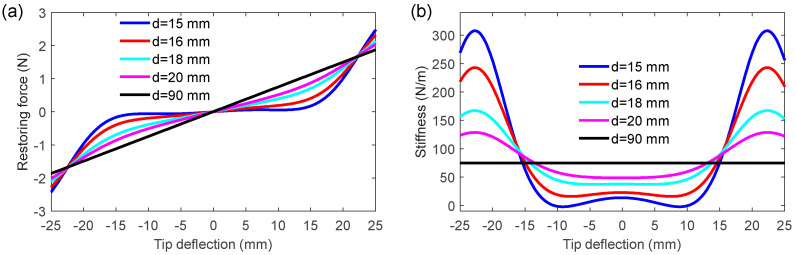
(**a**) Restoring force; (**b**) stiffness at various separation distances.

To illustrate the variation of the MPEH’s characteristics with respect to *d*, the restoring force and stiffness at different separation distances are depicted in Figure 4. It is obvious that the restoring force and stiffness are greatly reduced with a decrease in *d*. The reason is that the attractive magnetic force will keep increasing when *d* decreases and will reach nearly an equal value to the elastic force at a critical distance. The MPEH will have an extremely low restoring force and bending stiffness. Therefore, even under the weak vibration of the bridge, the large deflection of the MPEH is likely to happen, thereby producing a high-voltage output. In contrast, the linear harvester only has the satisfactory harvesting efficiency while the excitation frequency matches the resonance frequency. However, in the practical environment, the speeds and weights of vehicles passing the bridge usually exhibit stochastic characteristics. Therefore, the vibration energy generated by passing vehicles distributes over a fairly broad frequency band. Thus, in terms of practical excitation, the MPEH may be superior to the LPEH.

## 4. Experiment Setup

**Figure 5:**
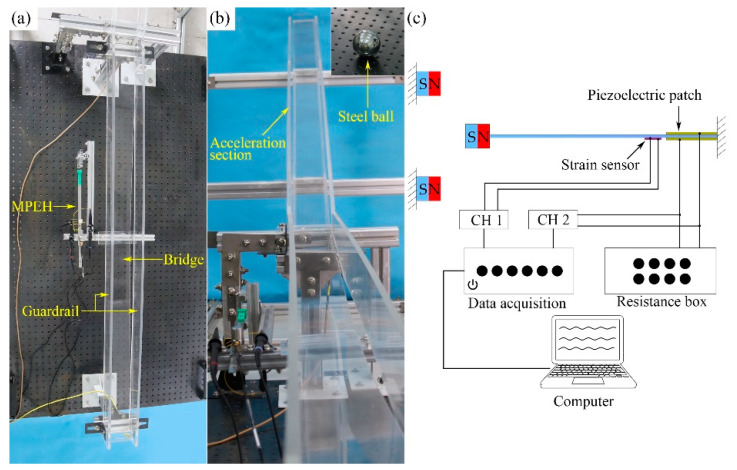
The experimental setup: (**a**) test section, (**b**) acceleration section, and (**c**) schematic diagram of equipment.

**Figure 6:**
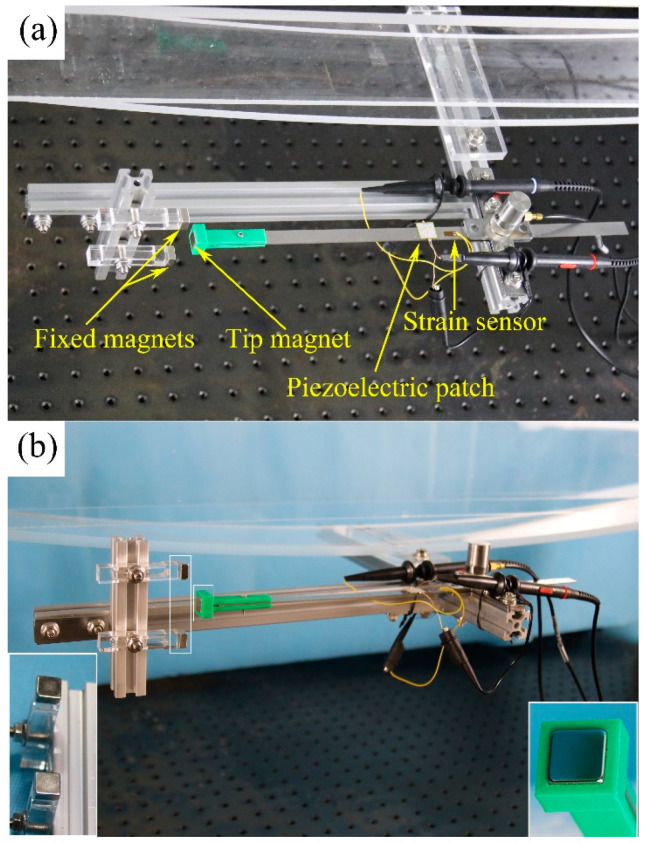
Prototype of the MPEH: (**a**) top view and (**b**) side view.

**Figure 7:**
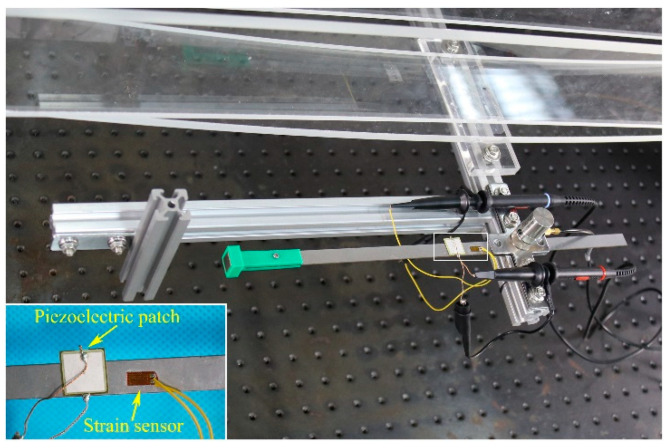
Prototype of the LPEH.

In order to show the dynamic response and performance of the electric output of the MPEH, a prototype of the MPEH is designed and fabricated. The experimental setup is shown in Figure 5. The model bridge is made from an acrylic sheet with the dimensions of 1300 mm × 130 mm × 8 mm (seen in Figure 5a). The moving vehicle is simulated by a moving steel ball with the mass of 1.003 kg, which can be accelerated from different heights of the acceleration track, as shown in Figure 5b. The guardrails are installed on both sides of the bridge to prevent the steel ball from falling off the bridge. The schematic diagram of the experiment equipment is presented in Figure 5c. The MPEH is fixed in the middle of the bridge, as shown Figure 6. The related parameter values are listed in Table 1. The Piezoelectric patch pasted onto the substrate of the MPEH is connected to a resistance box. The strain sensor (120-5AA) is attached to the substrate of the MPEH near the piezoelectric patch. When it passes the bridge, the moving load will excite the bridge to oscillate. As for the three magnets, one is attached to the free end of the piezoelectric beam, and the other two magnets are fixed at the fixture. The separation distance (d) and gap distance ($d_{g}$) are set to 17 mm and 45 mm, respectively. The LPEH is fabricated as well, as shown in Figure 7, which is similar to the MPEH in configuration but does not have the two fixed magnets. In the experiments, the LPEH and MPEH were put in the same testing environment for comparison. The strain response and voltage output of the harvester under bridge vibration were displayed and recorded by a digital signal acquisition device (DH5922N, Dong Hua). The value of the sampling frequency was set to 1000 Hz. In each test, to simulate different speeds of vehicles, the steel ball was released from different heights of the acrylic track.

## 5. Results and Discussions

**Figure 8:**
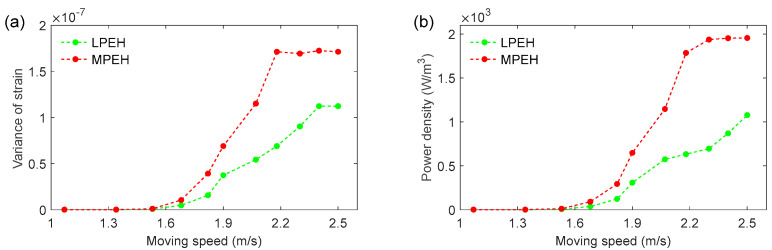
(**a**) Variance of strain; (**b**) power density of LPEH and MPEH for different moving speeds.

In the experiment, both the LPEH and MPEH were installed in the middle span of the bridge. A strain sensor and a piezoelectric patch were bonded to the root of the substrate so we could obtain the strain response of the system and the dynamic output voltage. Figure 8 gives the variance of strain and power density (W/m^3^) at the load resistance of 0.9 MΩ for different moving speeds. To control the moving speed, the steel ball was put on an inclined track and released at different heights. The running interval was controlled to be between 0.50 s and 1.07 s, corresponding to the practical interval of a vehicle passing through bridges. From Figure 8, it is clear that for both the LPEH and MPEH, their variances of strain and power density increase with the moving speed. Especially for the speeds higher than 1.82 m/s, the MPEH’s power density increases rapidly and outperforms that of LPEH, and so does the MPEH’s strain.

**Figure 9:**
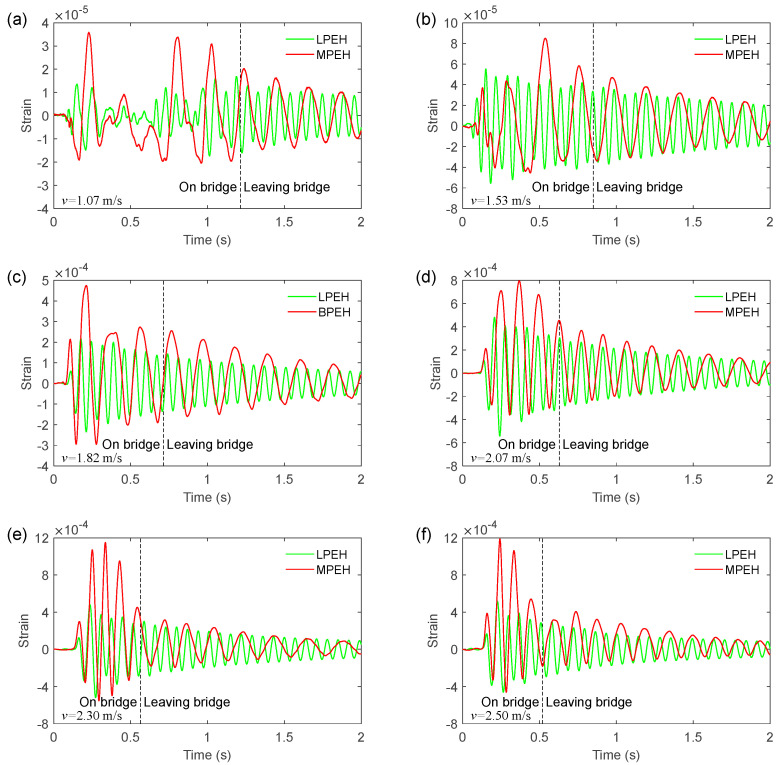
Strain responses of the LPEH and the MPEH for the vehicle travelling on and leaving the bridge: (**a**) *v* = 1.07 m/s, (**b**) *v* = 1.53 m/s, (**c**) *v* = 1.82 m/s, (**d**) *v* = 2.07 m/s, (**e**) *v* = 2.30 m/s and (**f**) *v* = 2.50 m/s.

To show the dynamics of the MPEH and LPEH, the time histories of strain are illustrated in Figure 9, in which six moving speeds (*v* = 1.07, 1.53, 1.82, 2.07, 2.30, and 2.50 m/s) are considered. In Figure 9, at *t* = 0 s, the vehicle arrives at the bridge, while the dashed line denotes the moment the vehicle leaves the bridge. The label “On bridge” represents the time interval of the vehicle moving on the bridge, while the label “Leaving bridge” represents the time interval after the vehicle leaves the bridge. In all cases, it is apparent that the strain of the MPEH is much larger than that of the LPEH when the vehicle travels on the bridge. This can be explained by the fact that an impact load acts on the bridge at the instant when the vehicle just enters the bridge. The MPEH is designed to have two magnets, with which the potential energy can be tailored to have a wide and flat bottom. This potential energy is beneficial for the occurrence of a large deflection. Thus, the small vibration of the bridge can trigger the large deflection of the MPEH, which is desirable in energy harvesting. Specifically, at a slow speed, e.g., *v* = 1.07 m/s, the maximum strain value of the MPEH can reach 3.6 × 10^−5^, which is 164% higher than that of the LPEH (1.36 × 10^−5^). In both the MPEH and LPEH, the maximum strain increases with the moving speed. In any case, the vibration period of the MPEH is much longer than that of the LPEH, which is beneficial to harvesting weak vibration energy. After the vehicle leaves the bridge, the responses of the LPEH and MPEH exhibit a characteristic of decay vibration. Although the amount of time that the vehicle moves on the bridge is not very long due to its relatively fast speed (such as *v* = 2.07 m/s, 2.30 m/s, or 2.5 m/s), the frequency and amplitude of a large strain is satisfactory. From the comparison of strains between the MPEH and LPEH, it can be concluded that the deflection of the MPEH is much larger than that of the LPEH, particularly during the period when the vehicle is moving on the bridge.

**Figure 10:**
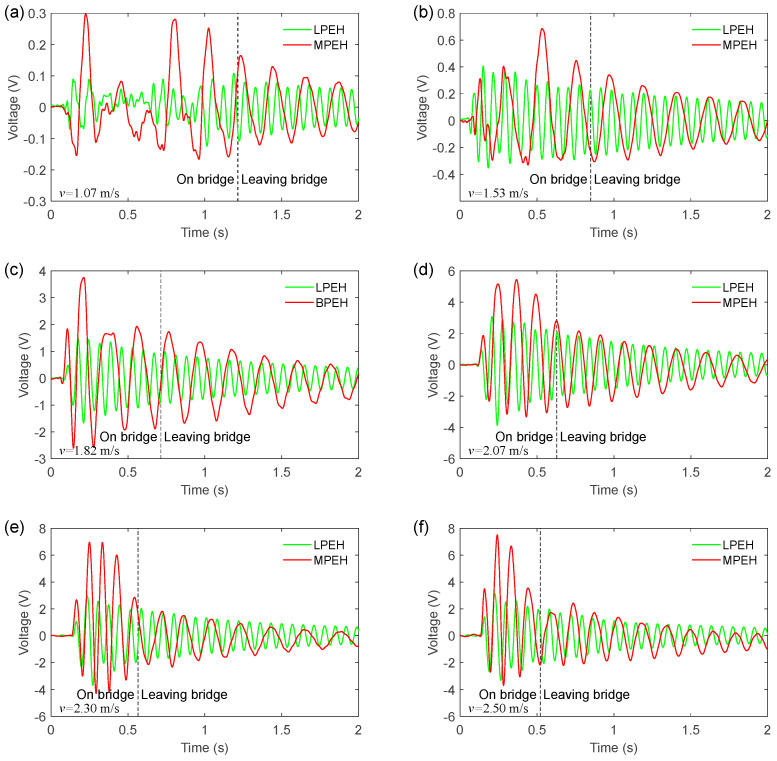
Voltage output of the LPEH and MPEH for the vehicle travelling on and leaving the bridge: (**a**) *v* = 1.07 m/s, (**b**) *v* = 1.53 m/s, (**c**) *v* = 1.82 m/s, (**d**) *v* = 2.07 m/s, (**e**) *v* = 2.30 m/s and (**f**) *v* = 2.50 m/s.

Figure 10 shows the open-circuit voltages of the LPEH and MPEH when the vehicle travels on and leaves the bridge. By comparing Figure 9 with Figure 10, it can be concluded that the deflection of the harvester is closely related to the output voltage. Moreover, from Figure 10, it can be seen that the voltage of the MPEH is significantly larger than that of the LPEH. In all cases, the large output voltage is generated during the period when the vehicle is travelling on the bridge, in which the vibration amplitude makes a major contribution to the voltage output. For a slower speed of 1.53 m/s, the voltage amplitudes of the LPEH and MPEH are 0.41 V and 0.57 V, respectively. The vibrations caused by the impact load and the moving vehicle are very weak, which make the LPEH execute a small amplitude vibration. For the MPEH, owing to the nonlinear magnetic forces, the vibration amplitude is relative large under weak excitations. Thus, the MPEH could improve the voltage output at slower speeds. By increasing the moving speed, the bridge vibration caused by the moving vehicle becomes violent. Thus, the amplitude of the voltage output increases accordingly, resulting in a high harvesting efficiency. It should be noted that when the speed of the vehicle reaches a certain level (such as 2.30 m/s or 2.50 m/s), there is no significant increase in the voltage amplitude. For example, the maximum voltage of the MPEH reaches 6.94 V for the speed of 2.30 m/s. However, as the moving speed increases to 2.50 m/s, the maximum voltage only has a small increase and reaches 7.49 V. This is because the MPEH’s two magnets are prompted to protect the piezoelectric material from damage when the beam deflection is too large.

**Figure 11:**
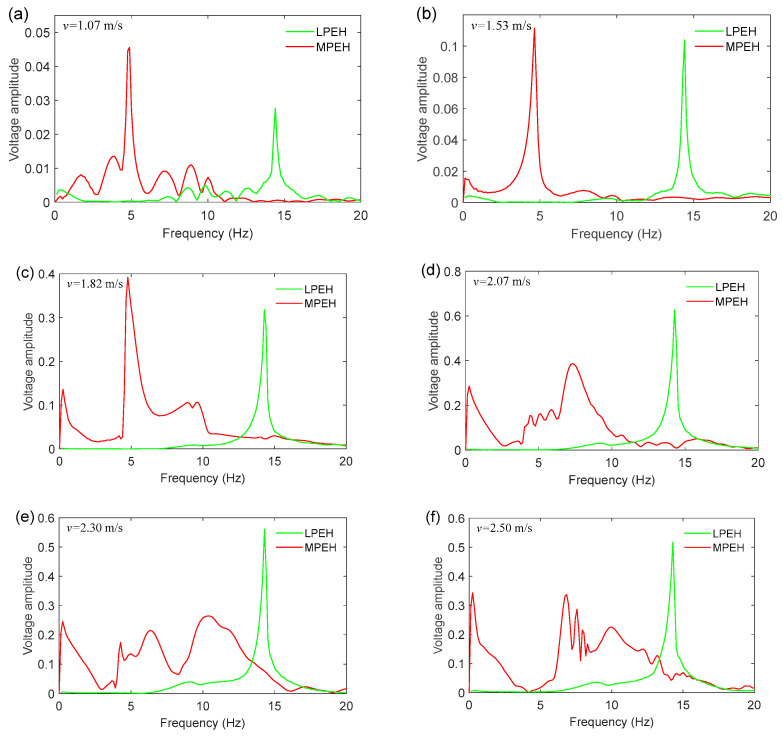
Frequency spectra of the output voltage for a vehicle travelling on and leaving the bridge: (**a**) *v* = 1.07 m/s, (**b**) *v* = 1.53 m/s, (**c**) *v* = 1.82 m/s, (**d**) *v* = 2.07 m/s, (**e**) *v* = 2.30 m/s and (**f**) *v* = 2.50 m/s.

In order to better understand the dynamic characteristics of the LPEH and MPEH, their frequency spectra of output voltage are shown Figure 11. The peak of the LPEH is located near 14.5 Hz. In contrast, the MPEH’s peak changes with the vehicle speed. For different moving speeds, unlike the LPEH, the MPEH has a different frequency spectrum distribution. More specifically, at *v* = 1.07 m/s, the frequency spectrum of the MPEH output is located at 4.90 Hz, and the voltage amplitude of the MPEH is significantly greater than that of the LPEH. As the moving speed increases to 2.07 m/s, the main peak of voltage shifts to 7.32 Hz, and the response energy mainly distributes over a wide frequency range of 0–10.0 Hz (Figure 9d). It should be noted that a peak appears at the extremely low frequency of 0.25 Hz, corresponding to the motion between the two fixed magnets or to the high-energy orbit. Then, by further increasing the moving speed, the frequency distribution of the voltage is changed, but the peak at the extremely low frequency increases steadily, implying that the response between the two fixed magnets enhances gradually. In particular, at *v* = 2.30 m/s and *v* = 2.50 m/s, from the frequency spectra of the voltage, it is apparent that the vibration energy is concentrated in the range of 0–15 Hz, with a peak of the extremely low frequency, as shown in Figure 9 and Figure 10 (the vibration period of the MPEH is significantly larger than that of the LPEH). Therefore, for the MPEH, since the potential energy is controlled to have an optimal shape, the energy required to produce a large deflection is greatly reduced due to a comparatively strong coupling between the elastic force and the magnetic force. Especially in practice, the moving vehicles consecutively travel on a bridge, so the MPEH will be excited continuously. Then, the MPEH can keep oscillating in a high-energy orbit and produce a consecutive large output. Thus, the MPEH is suitable for harvesting vibration energy from a practical traffic bridge.

## 6. Conclusions

In summary, this paper reports a novel concept on how to improve energy harvesting from traffic bridges. The magnets are introduced to tailor the potential energy rather than to create bi-stability. By adjusting the magnets’ positions, the ideal mono-stable configuration with an extremely low restoring force and bending stiffness can be obtained. This desirable configuration can make the beam oscillate in a high-energy orbit and gives a large output under weak bridge excitations. The experimental results show that the energy harvesting performance of MPEH is significantly higher than the LPEH when the vehicle speed exceeds 1.82 m/s. For different moving speeds, the MPEH has an extreme peak in frequency spectrum, corresponding to a high-energy orbit. This study may open a new way for energy harvesters designed for traffic bridges. However, some further investigations are still needed to maximize the energy harvesting output. The separation distance and gap distance are two key factors for potential energy, the restoring force, and stiffness. The performance of the MPEHs can be promoted further by optimizing the tip magnet and the fixed magnet.

## Figures and Tables

**Table 1:** Properties for the analysis of potential energy function.

Symbol	Parameter	Value
Substrate		
$L_{s}$	Length	190 mm
$T_{s}$	Thickness	1 mm
$W_{s}$	Width	10 mm
$E_{s}$	Young modulus	205 GPa
Piezoelectric patch		
$L_{p}$	Length	5 mm
$T_{p}$	Thickness	5 mm
$W_{p}$	Width	0.25 mm
$E_{p}$	Young modulus	56 GPa
Magnet		
$a_{1}$$(a_{2}$$,\;a_{3}$)	Effective magnetic moment	0.218 Am^2^
$d_{g}$	Gap distance between the two fixed magnets	45 mm
$\mu_{0}$	Permeability constant of the magnet	4 × 10^−7^ NA^−2^

## Data Availability

The data presented in this study are available on request from the corresponding author.
